# Percent positivity and phylogenetic analysis of *Mycoplasma gallisepticum* and *Mycoplasma synoviae* in commercial poultry from the different States of India

**DOI:** 10.14202/vetworld.2022.1843-1851

**Published:** 2022-07-28

**Authors:** Pranoti Giram, Pankhudi Bhutada, Chhagan Prajapati, Santosh S. Koratkar, Sachin Patil, Devender Hooda, Vinay Rale, Satish S. Tongaonkar

**Affiliations:** 1 Symbiosis School of Biological Sciences, Symbiosis International (Deemed University), Lavale, Maharashtra, India; 2 Huvepharma SEA (Pune) Pvt. Ltd., Maharashtra, India; 3 Symbiosis Centre for Research & Innovation (SCRI), Symbiosis International Deemed University, Lavale, Pune 412115, Maharashtra, India

**Keywords:** epidemiology, India, *Mycoplasma gallisepticum*, *Mycoplasma synoviae*, phylogeny, polymerase chain reaction

## Abstract

**Background and Aim:**

The Indian and global poultry industries suffer significant economic losses due to *Mycoplasma gallisepticum* (MG) and *Mycoplasma synoviae* (MS) infections, which adversely affect egg production, hatchability, weight gain, and feed efficiency in farms, thus decreasing the overall production efficiency. This study aimed to determine the percent positivity and phylogenetic analysis of MG, MS, and co-infection of both mycoplasmas in commercial poultry farms across different states of India from 2017 to 2021.

**Materials and Methods:**

A total of 3620 tracheal or choacal swabs were collected from breeder and layer farms showing clinical signs of avian mycoplasma infections from commercial poultry farms across India, and the percent positivity for MG, MS, and co-infection of both mycoplasmas were determined by Polymerase chain reaction using the *16S rRN*A and *vlhA* genes amplification, respectively. Phylogenetic analysis was carried out by sequencing the *mgc2* and *vlhA* genes of 2 samples of MG and 24 samples of *M. synoviae* to gain insight into the genetic variability of Indian strains. The data were then compared with other Indian strains, vaccines strains, and strains from other countries.

**Results:**

Our data shows the percent positivity of MG, MS, and co-infection of both MG and MS was 6.43%, 23.61%, and 15.49%, respectively. The phylogenetic relationship between MG and MS was determined using the *vlhA* and *mgc2* genes, revealing two samples of MG and 24 samples of MS clustered with other Indian strains. *M. synoviae* MSM22 and previously studied *M. synoviae* MGS 482 clustered with vaccine strain *M. synoviae* MS-H.

**Conclusion:**

*Mycoplasma synoviae* infections in breeder, layer, and in both is predominant compared to MG across the states investigated in India. Sequenced samples of MG and MS showed evolutionary relationships with the previously studied Indian strains of MG and MS. These findings support our view that monitoring chickens for avian mycoplasma infections are of paramount significance. It further lends credence to the contention that such information will pave the way for the development of a home-grown vaccination control program and thus safeguard the poultry sector against mycoplasma infections.

## Introduction

Mycoplasma is a common avian bacterial pathogen causing enormous economic losses to the poultry industry worldwide. *Mycoplasma gallisepticu*m (MG) and *Mycoplasma synovia*e (MS) are the most important species causing chronic respiratory disease and acute synovitis in birds. MG causes acute and chronic respiratory infections of the upper respiratory tract with symptoms such as coughing, nasal and ocular discharge with poor productivity, slow growth, reduced hatchability and viability, and occasional encephalopathy, eventually leading to colossal losses [1–3]. MS causes acute to chronic air sacculitis by infecting the mid-lower respiratory tract and air sacs, as well as eggshell apex abnormalities and drop-in egg production. Inflammation of synovial membrane, joints, and tendon sheaths is a consequence of systemic MS infection [1, 2, 4]. The infections associated with these two pathogens persist in poultry due to horizontal and vertical transmission [2]. Co-infection with Newcastle disease virus (NDV), infectious bronchitis virus (IBV), and avian pathogenic *Escherichia coli* (APEC) can further increase the impact of an outbreak of mycoplasmas. Antibiotic treatment and vaccination are routine practices in India to surmount *Mycoplasma* infections in poultry [5].

Prior prevalence studies have shown the presence of *Mycoplasma* infections in different parts of India. For example, 24 samples investigated from flocks with eggshell apex abnormalities in Tamil Nadu manifested MS (16.6% of the samples) and MG and MS together (12.5% of the samples) [6]. A countrywide mycoplasma prevalence study was performed with a two-test approach using polymerase chain reaction (PCR) analysis and enzyme-linked immunosorbent assay(ELISA) on 635 samples. The PCR results showed 33% of samples positive for MS and 11.7% for MG whereas the ELISA yielded 52.1% positive for MS and 32.6% positive for MG, indicating the dominance of MS in India [5].

This study aimed to determine the percent positivity and phylogenetic analysis of MG, MS, and co-infection of both mycoplasmas in commercial poultry farms across different states of India from 2017 to 2021.

## Materials and Methods

### Ethical approval and Informed consent

Ethical approval is not required for this type of study. The broiler and layer breed/chickens were handled carefully by veterinary experts during tracheal or choanal swab collection. Prior verbal consent was obtained from the owner of each farm. The privacy and confidentiality of participating poultry farms and farm owners’ personal information is not disclosed in this study.

### Study period and location

The study was conducted from January 2017 to December 2021. Tracheal or choanal swabs of breeder and layer chickens were collected from different commercial poultry farms of Andhra Pradesh, Bihar, Goa, Gujrat, Haryana, Jharkhand, Karnataka, Kerala, Maharashtra, Odisha, Punjab, Rajasthan, Tamil Nadu, Telangana, Tripura, and West Bengal. The samples were processed at Symbiosis School of Biological Sciences, SIU, Pune, India.

### Sample collection

A total of 3,620 tracheal or choanal swabs were collected. Of these 2,576 samples originated from the breeders while 1,044 samples came from layers. A total of 231 poultry farms contributed samples. Chickens showing clinical signs which included tracheal rales, nasal discharge, coughing, reduced diet, weight loss, a decline in egg production, retarded growth, lameness, ruffled feathers, infectious synovitis along with blueish red comb, and breast blisters were suggestive of *Mycoplasma* [2]. Samples were collected by trained veterinary doctors who assessed the birds for clinical signs. The farm size of breeder chicken was about 10,000–100,000 birds and that of the layer farms had a room for 50,000–1000,000 birds. Each farm was separated into multiple sheds with capacities ranging from 5,000 to 10,000 birds. Sample collection was performed by purposive sampling method, having two male and three female samples from each shed. The samples were immediately transported to the laboratory on ice (4°C), maintaining a cold chain. The samples were collected in batches and processed accordingly.

### Sample processing and genomic DNA extraction

The collected tracheal or choanal swabs were resuspended in 600 mL of 1× phosphate-buffered saline in batches of 10–30 samples. The genomic DNA was isolated using the standard phenol-chloroform-isoamyl alcohol method [7]. The quantity and quality of genomic DNA was estimated using NanoDrop™ 2000 Spectrophotometer (Thermo Fisher Scientific, Waltham, MA, USA).

### PCR

MG-14F and MG-13R [8] and MSlink-F and MScons-R [9] primers (Bioserve Biotechnologies (India) Pvt. Ltd., India) were used for the detection of the current positivity study while mgc2-1F and mgc2-1R [2, 10] and MSlink-F and MScons-R [9] primers (Bioserve Biotechnologies) were used for phylogenetic analysis of MG and MS, respectively (Table-1) [2, 8–10]. The *16S rRNA* gene of MG and *vlhA* gene of MS were amplified using multiplex PCR in Applied Biosystems™ 2720 Thermal Cycler (Thermo Fisher Scientific). The thermal cycling conditions were as follows: initial denaturation at 94°C for 30 s; followed by 35 cycles of denaturation at 94°C for 30 s, primer annealing at 55°C for 30 s, extension at 72°C for 1 min, and a final extension at 72°C for 7 min. A phylogenetic analysis was performed by amplification of *mgc2* gene of MG using primers mgc1F and mgc1R [2]. PCR products were electrophoresed (Bio-Rad Laboratories, USA) on a 1.5% agarose gel (HiMedia, India) in ×1 Tris-acetic acid-Ethylenediaminetetraacetic acid buffer (Sigma-Aldrich, Bulgaria) at a constant voltage of 80 V, stained with tracking dye ethidium bromide (10 mg/mL) (Sigma-Aldrich), visualized under ultraviolet light by gel documentation (GelDoc EZ Gel Documentation System, Bio-Rad Laboratories), photographed and recorded for target gene amplification (*16S rRNA* gene - 187 bp, *vlhA* - 300–400 bp, and *mgc2* - 824 bp).

**Table 1:** Sequences of 16S rRNA, vlhA, and mgc2 gene primers and sizes of amplicon used for the detection of *Mycoplasma gallisepticum and Mycoplasma synoviae*.

Organism	Gene name	Primers	Sequence	Amplicon size (bp)	Reference
*Mycoplasma gallisepticum*	*16S rRNA*	MG-14F	5’-GAGCTAATCTGTAAAGTTGGTC-3’	187 bp	[8]
		MG-13R	5’-GCTTCCTTGCGGTTAGCAAC-3’		
*Mycoplasma synoviae*	*vlhA*	MS-linkF	5’-TACTATTAGCAGCTAGTGC-3’	300–400 bp	[9]
		MS-consR	5’-AGTAACCGATCCGCTTAAT-3’		
*Mycoplasma gallisepticum*	*mgc2*	mgc2-1F	5’-GCTTTGTGTTCTCGGGTGCTA-3’	824 bp	[2 10]
		mgc2-1R	5’-CGGTGGAAAACCAGCTCTTG-3’		

### Percent positivity of MG and MS

A percent positivity was calculated for breeder, layer and both year wise and state wise from 2017 to 2021. The positivity rate was examined for only MG, only MS, and co-infection of both mycoplasmas for different states across different states of India.

### Statistical analysis

The average percent positivity of samples calculated for bird type and year-wise was further categorized into various groups MG vs. MS, MG vs. MG + MS, and MS vs. MG + MS and was compared using Student’s t-test (two-tailed, type 2), average percent positivity was meditated (single or co-infection) ± standard deviation and differences were considered significant for p < 0.05*, p < 0.01**, and p < 0.001***.

### Sanger sequencing and analysis

The amplified PCR products for MG *mgc*2 gene and M*S vlhA* gene were purified using HiPurA™ Quick gel purification kit (HiMedia). The purified PCR product of two samples of MG and 24 samples of MS were sequenced by Applied Biosystems 3130 Genetic Analyzer (Hitachi, Japan). Sequence editing, alignment, and assembly were performed with SeqMan Pro modules of the DNASTAR^®^ Lasergene Software v14 (DNAStar, Madison, WI, USA) [11]. BLAST searches were performed using the Nucleotide Basic Local Alignment Search Tool (BLASTn) algorithm to compare sequences with those in the National Center for Biotechnology Information (NCBI) database (http://ncbi.nlm.nih.gov) and all sequences of MG and MS were submitted to NCBI under the accession numbers MZ079374 to MZ079399.

### Phylogenetic analysis

The sequences of MG (MGH01 and MGM02), MS (MSM01 to MSM24), reference strains from different countries and vaccine strains were aligned by Multiple Sequence Comparison by Log-Expectation (MUSCLE) [11, 12]. The best model for sequence data was predicted, rooted, and constructed in MEGA version 7.0 for evolutionary study [13–15]. The topologies of the phylogenetic trees were assessed by bootstrap values determined based on 1000 replications. The phylogenetic tree of MG is rooted with MG strain F and MS is rooted with MG strain K2966 (US) and MG strain K6112B-8 (US).

## Results

**Figure-1:**
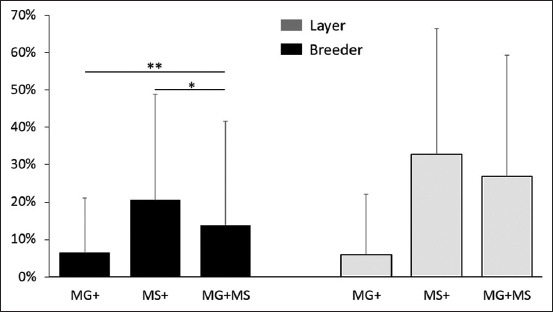
Average percent positivity of MG, MS, and co-infection (MG + MS) in breeder and layer from 2017 to 2021. MG average percent positivity was calculated for samples showing only positive for MG, similarly, for MS and MG + MS represent co-infected with both. Each bar represents average percent positivity (single or co-infection) ± standard deviation and differences were considered significant for p < 0.05*, p < 0.01**, and p < 0.001***.

**Figure-2:**
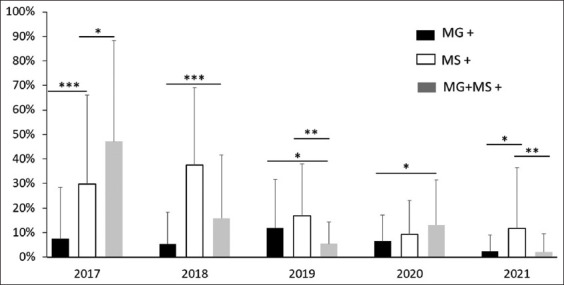
Year-wise percent positivity of MG, MS, and co-infection (MG + MS) in breeders and layers from 2017 to 2021. Each bar represents average percent positivity (single or co-infection) ± standard deviation and differences were considered significant for p < 0.05*, p < 0.01**, and p < 0.001***.

The number of samples undertaken for detection of MG and MS is given in Table-2. The percent positivity of MG, MS, and co-infection (MG + MS) in breeder and layer is shown in Figure-1 and the year-wise percent positivity of MG, MS, and co-infection (MG + MS) in breeders and layers is given in Figure-2.

**Table 2:** Overall percent positivity of MG, MS, and co-infection (MG+MS) in breeder and layer from 2017 to 2021.

Type of bird	MG (%)	MS (%)	MG-MS (%)	Total (%)
Breeder	64 (6.13)	341 (32.66)	287 (27.49)	1044 (66.28)
Layer	169 (6.56)	514 (19.95)	274 (10.63)	2576 (37.12)
Total	233 (6.44)	855 (23.61)	561 (15.49)	3620

### Percent positivity of *M. gallisepticum* and *M. synoviae* in different states

From the total number of samples, the percent positivity of MS was 23.61%, higher than MG (6.43%) and co-infection of both mycoplasmas (15.49%). The same pattern was demonstrated in both breeders and layers as presented in Table-2. When the total number of samples was compared amongst breeders, it was more significant in MG versus MG + MS, than MS versus MG + MS as shown in Figure-1. When positivity in layers was compared, there was no significant difference when MG versus MS or MG versus MG + MS and MS versus MG + MS were compared (Figure-1).

**Figure-3:**
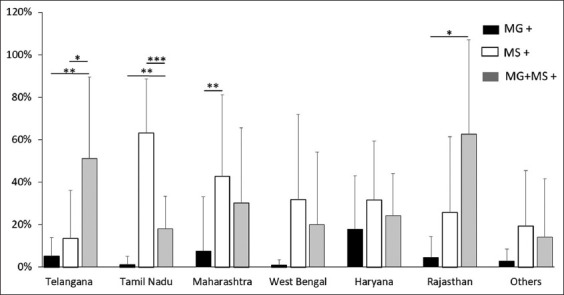
State-wise average percent positivity of MG, MS, and co-infection (MG + MS) from 2017 to 2021 in layers. Each bar represents average percent positivity (single or co-infection) ± standard deviation and differences were considered significant for p < 0.05*, p < 0.01**, and p < 0.001***.

**Figure-4:**
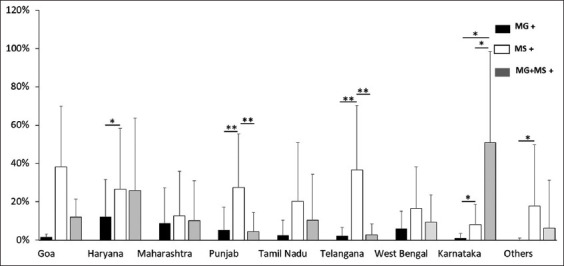
State-wise average percent positivity of MG, MS, and co-infection (MG + MS) from 2017 to 2021 in breeders. Each bar represents average percent positivity (single or co-infection) ± standard deviation and differences were considered significant for p < 0.05*, p < 0.01**, and p < 0.001***.

**Figure-5:**
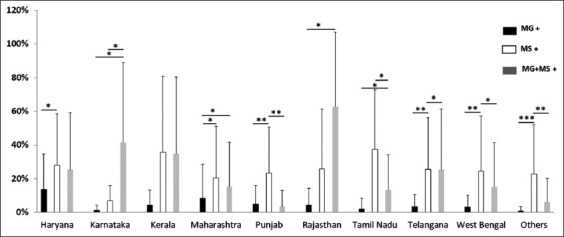
State-wise average percent positivity of MG, MS, and coinfection (MG + MS) from 2017 to 2021 in commercial poultry farms (breeder and layer) of India. Each bar represents average percent positivity (single or co-infection) ± standard deviation and differences were considered significant for p < 0.05*, p < 0.01**, and p < 0.001***.

In the case of layer birds, evaluation of the average percent positivity of MG, MS, and co-infection, revealed that the positivity of MS and co-infection was greater than MG in all investigated states. MS positivity was greater than co-infection positivity in Tamil Nadu, Maharashtra, West Bengal, Haryana, and other states, whereas co-infection positivity was higher in Telangana and Rajasthan (Figure-3). A similar pattern of MG positivity with MS and co-infection was also observed in the case of breeder birds. Except for Karnataka, all states had greater levels of MS positivity. MS positivity was substantially greater in Haryana, Panjab, Telangana, Karnataka, and other states than in MG (Figure-4). Further a combined graph of breeder and layers was plotted and analyzed. MS positivity was greater in all states except Karnataka and Rajasthan (Figure-5). The significance of the positivity of MG, MS and co-infection is represented in Figure-5. Overall, the results indicate MS dominance over MG and co-infection, with the co-infection positivity on the upper side.

### Phylogenetic analysis for *M. gallisepticum* and *M. synoviae*

**Figure-6:**
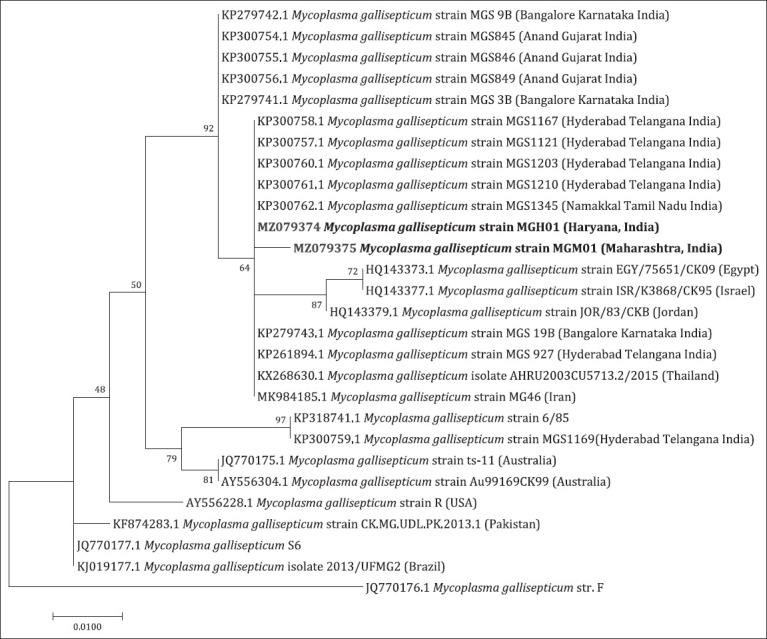
The evolutionary history of the mgc2 gene was inferred using the Maximum Likelihood method based on the Kimura 2-parameter model with 1000 bootstrap were conducted in MEGA7.

**Figure-7:**
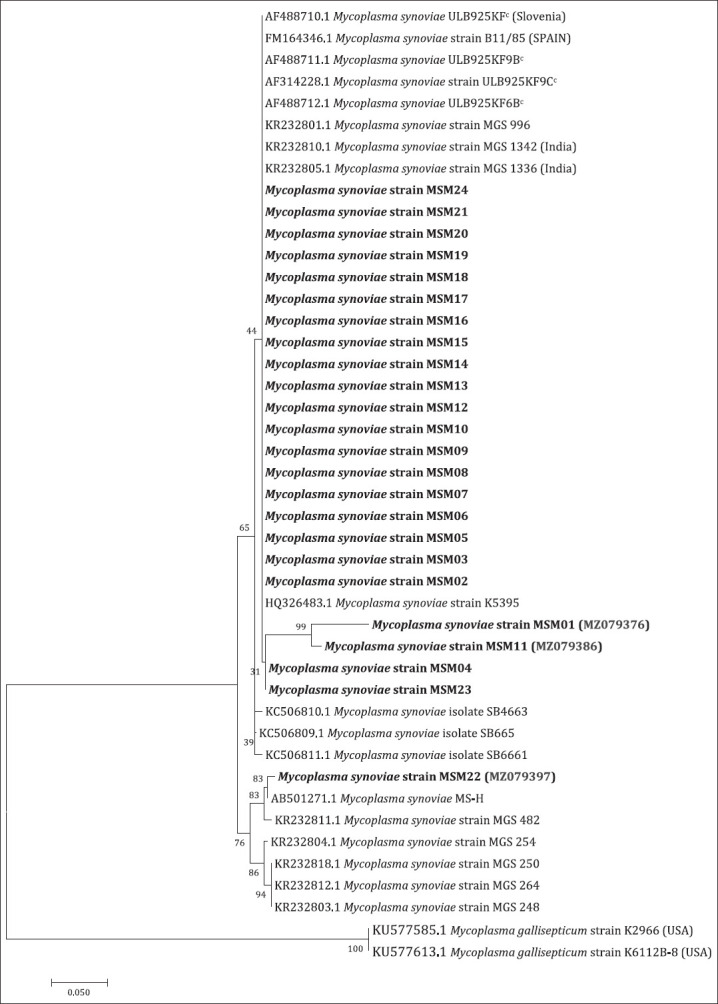
The evolutionary history of the vlhA gene was inferred using the Maximum Likelihood method based on the Hasegawa-Kishino-Yano model with 1000 bootstrap were conducted in MEGA7.

The *mgc2* gene of two MG samples and *vlhA* gene of twenty-four MS samples were sequenced from Maharashtra and Haryana. MG samples, MGH01 (Haryana) and MGM01 (Maharashtra), were closely related to previously documented Indian strains of MG. Both of the sequenced samples were distinctly different from the vaccine strains (ts-11, 6/85, and S6) of MG demonstrated in Figure-6. As per Figure-7, MSsamples MSM01, MSM11, MSM04, and MSM23 formed a separate sub-cluster within a large cluster containing almost all sequenced samples and other strains from India, United States, Slovenia, and Spain. The MSM22 was closely related to MSMS-H (vaccine strain from Australia) and MS strain MGS 482 (field isolate from India).

## Discussion

The increasing incidence of avian mycoplasma infections has led to substantial economic losses worldwide [2]. Chronic respiratory disease caused by MG contributes to 11.65% of mortality in chickens indicating high positivity among Indian poultry, whereas MS causes infectious synovitis and subclinical upper respiratory tract infection contributing to 33% of mortality among chickens [5]. To understand the epidemiology of these two pathogens, PCR and sequencing were performed. Phylogenetic analysis revealed the genetic diversity, transmission of infection, and differentiated them from the vaccine strain. PCR is considered a reliable, rapid, and accurate tool for the early detection of MG and MS from clinical specimens [2]. In the present study, 3620 samples tested for the presence of MG and MS, 381 samples showed 28.3% positivity for MG among breeders in Haryana, whereas 68 samples showed 17.8% positivity for co-infection. A previous study from Haryana [16] tested 92 samples by PCR and documented 27% positivity for MG. A study by Rajkumar *et al*. [5] for 309 samples showed 11.6% positivity for MG and 33.0% positivity for MS among 26 breeder flocks in Haryana. MS was noted as second-highest among the mentioned data for breeders of Haryana, India. In contrast, a study demonstrated the high prevalence of MG infection in commercial breeder farms of Haryana [16]. Out of 143 samples from Goa 21 were positive for MG, 74 were positive for MS, and 18 had co-infection (Figure-4). MG and MS infection among breeder chicken were noted for the first time in Goa, India. The percent positivity rate in the present study of MG and MS was 20.66% and 25.63% obtained in Maharashtra in breeders, whereas in an earlier reported study by Bagal [17], the percent positivity observed was 10% and 22% for MG and MS, respectively. These comparative analyses represent a similar pattern of infectivity.

MG and MS infections in layers start with egg drops and eventually leads to immunosuppression which makes them more susceptible to other diseases [2]. As per a worldwide pooled epidemiological meta-analysis, percent positivity for MG was 17.75% (3935/22162), and MS 74.89% (19783/26413) was reported. Meta-analysis data of India MG 16.25% (151/929) and MS 50.74% (68/134) was comparable with present study MG 21.93% (794/3620), MS 39.11% (1416/3620) [18]. Molecular study conducted by Prajapati *et al*. [19] in different countries indicate an increase in the prevalence of MG and MS infections among breeder and layer chickens. Malaysia, Bangladesh, and Belgium, including India, have reported a higher incidence of infections for co-infection in breeder and layer chickens. A study by Prajapati *et al*. [19] reported prevalence in breeder and layer chickens primarily for MG than MS, and co-infection. This study, reported that the percent positivity was higher in MS among breeder and layer chickens from various states in India. Another unique finding was the detection of co-infection among the breeder and layer, suggesting that co-infection in breeders and layers, if they go undetected/untreated, can result in huge losses.

The 16S rRNA sequence was not used for phylogenetic analysis because it has a highly conserved region among *Mycoplasma* species; therefore, the phylogenetic analysis of these sequences is not of much importance [20]. The phylogenetic analysis based on *mgc2* of MG samples MGH01 and MGM01 were clustered with previously documented Indian sequences MG [18, 19]. In a similar kind of study, the Indian field isolates of MG shared 90–100% identity with strains isolated in Thailand and Israel [5], while one strain shared 100% identity with the MG vaccine strain (MG 6/85) [21].

Out of 24 sequences of MS analyzed for *vlhA* gene study, most were grouped with previously documented Indian isolates of MS. Four samples, namely, MSM01 (MZ079376), MSM11 (MZ079386), MSM04 (MZ079379), and MSM23 (MZ079398), formed separate sub-clades within the clade that held most of the MS samples from this study. MS sample MSM22 (MZ079397) was closely related to MS MS-H (MS vaccine strain, Australia) and formed a separate clade with that strain along with a previously reported Indian isolate MS strain MGS 482 (India) [21]. Overall, this study has led to vital outcome into the diversity and distribution of MS in different states in India. This will necessitate continuous epidemiological investigations for the prevention and control of MS infection.

Since the positivity of MS was higher than that of MG among both breeders and layers; strategies must be devised to control MS infection in poultry. Epidemiological, genetic variability, and diversity studies must also be undertaken regularly for further information regarding MG and MS infections in India.

## Conclusion

This PCR-based study provides significant insights into the infection status and expansion of MG and MS affecting the poultry industry. MS infection in breeder, layer, and in both is predominant compared to MG in the states that were included. Sequenced samples of MG and MS in this study demonstrated an evolutionary relationship with the previously studied Indian strains of both. *M. synoviae* MSM22 and previously reported *M. synoviae* MGS 482 show similarity with the vaccine strain (*M. synoviae* MS-H) of MS. The obtained data demonstrate the utility of monitoring chickens and could help to develop indigenous vaccines to control and prevent mycoplasma infections in the poultry industry. In the current investigation, samples were obtained from representative Indian states with a significant chicken population. It would be preferable in future research to include all or most of the Indian states. Other bird species such as ducks, quails, and turkeys should be included in epidemiological investigations in addition to chickens. The phylogenetic analysis of *mgc2* and *vlhA* gene is useful in studies of molecular epidemiology for MG and MS. Percent positivity and phylogenetic analysis of MG and MS denoted the limitation in current control programs, highlighting the urgent need to implement improved strategies that include more sensitive diagnostic methodologies and more efficient vaccines. The use of molecular methods is recommended to be widened for successful detection and surveillance of MG and MS infections in India. If similar studies to be conducted on a larger scale in the rest of the states of India, a better understanding of the prevalent strains of MG and MS would would emerge. Naturally, this data will immensely help in designing vaccine strategies for the control and prevention measures.

## Authors’ Contributions

SSK and SP: Conceptualization, planning, designing, supervision, analysis of data, and manuscript reviewing and proofreading. PG: Conducted experiments, collected data, performed the statistical analysis, Sanger sequencing, analysis of sequenced data, and wrote the original draft. CP: Processed PCR samples for detection of MG and MS. PB: Drafted and reviewed the manuscript. SP and DH: Identification and selection of farms, helped in sample collection, and recording of clinical and farm data. VR and SST: Reviewed and corrected the manuscript. All authors contributed to the preparation and revision of the manuscript. All authors have read and approved the final manuscript.
